# Identification and analysis of miRNAs in human breast cancer and teratoma samples using deep sequencing

**DOI:** 10.1186/1755-8794-2-35

**Published:** 2009-06-09

**Authors:** Sanne Nygaard, Anders Jacobsen, Morten Lindow, Jens Eriksen, Eva Balslev, Henrik Flyger, Niels Tolstrup, Søren Møller, Anders Krogh, Thomas Litman

**Affiliations:** 1 The Bioinformatics Centre, Department of biology, University of Copenhagen, 2200 Copenhagen N, Denmark; 2 The Biotech Research and Innovation Centre (BRIC), Department of biology, University of Copenhagen, 2200 Copenhagen N, Denmark; 3 Laboratory of Oncology, Herlev University Hospital, 2730 Herlev, Denmark; 4 Department of Pathology, Herlev University Hospital, 2730 Herlev, Denmark; 5 Department of Breast Surgery, Herlev University Hospital, 2730 Herlev, Denmark; 6 Exiqon A/S, Bygstubben 9, 2950 Vedbæk, Denmark; 7 Santaris Pharma A/S, Bøge Allé 3-5, 2970 Hørsholm, Denmark

## Abstract

**Background:**

MiRNAs play important roles in cellular control and in various disease states such as cancers, where they may serve as markers or possibly even therapeutics. Identifying the whole repertoire of miRNAs and understanding their expression patterns is therefore an important goal.

**Methods:**

Here we describe the analysis of 454 pyrosequencing of small RNA from four different tissues: Breast cancer, normal adjacent breast, and two teratoma cell lines. We developed a pipeline for identifying new miRNAs, emphasizing extracting and retaining as much data as possible from even noisy sequencing data. We investigated differential expression of miRNAs in the breast cancer and normal adjacent breast samples, and systematically examined the mature sequence end variability of miRNA compared to non-miRNA loci.

**Results:**

We identified five novel miRNAs, as well as two putative alternative precursors for known miRNAs. Several miRNAs were differentially expressed between the breast cancer and normal breast samples. The end variability was shown to be significantly different between miRNA and non-miRNA loci.

**Conclusion:**

Pyrosequencing of small RNAs, together with a computational pipeline, can be used to identify miRNAs in tumor and other tissues. Measures of miRNA end variability may in the future be incorporated into the discovery pipeline as a discriminatory feature. Breast cancer samples show a distinct miRNA expression profile compared to normal adjacent breast.

## Background

MicroRNAs (miRNAs) have rapidly emerged as an important class of short endogenous RNAs that act as post-transcriptional regulators of gene expression by base-pairing with their target mRNAs. The approximately 22 nucleotides (nt) long mature miRNAs are processed sequentially from longer hairpin transcripts by the RNAse III ribonucleases Drosha [1] and Dicer [2,3]. To date more than 9539 miRNAs have been annotated in vertebrates, invertebrates and plants of which 706 are human according to the miRBase database release 13.0 in March 2009 [4,5], and recent bioinformatic predictions combined with array analyses, small RNA cloning and Northern blot validation indicate that the total number of miRNAs in vertebrate genomes is significantly higher than previously estimated and may be thousands [6-8].

Several papers have already described the usefulness of miRNAs as diagnostic molecules in e.g. cancer [9,10] and their potential as therapeutics is being explored [11]. One of the obvious and important goals for understanding more precisely the role and importance of miRNAs in different cellular contexts is to identify all_miRNA species of a given organism and their expression profiles. The diminishing costs of High-Throughput (HT) sequencing techniques are making these increasingly more popular for such discovery and profiling efforts [12,13]. In consequence, large amounts of data will be generated, and appropriate bioinformatics methods are needed to deal with the data.

We developed a pipeline combining exact and probabilistic methods to analyse 454 small RNA data for the purpose of identifying putative new miRNAs. This task can be divided into two objectives: finding and quantifying expressed genomic regions giving rise to small RNA reads, and scoring these regions as potential new miRNAs. Our approach to the first part of this problem was to retain as much sequence information as possible, despite possible sequencing errors and redundant mapping, thus increasing the amount of available data. For the second objective, we trained a Support Vector Machine (SVM) for reliable classification of potential miRNAs.

The pipeline was used to analyze deep sequencing data generated from four different human tissue samples: Breast cancer, normal adjacent breast, and two teratoma cell lines. We chose to analyze breast cancer associated miRNAs, as these represent an important case for finding miRNA based biomarkers for cancer diagnosis. The discovery of novel miRNAs, as well as understanding the expression of already known miRNAs in these tissues, is therefore of medical interest. The two teratoma cell lines were included in the analysis with the aim of identifying novel miRNAs. Given that teratoma can develop into many different tissue types, we hypothesized that these samples could potentially express different miRNAs than normal samples and thus be a good source of new miRNAs.

In several papers it has been observed that the 5' end of metazoan mature miRNAs is more precisely defined than the 3' end [14-17]. Recently Seitz *et al*. reported the first systematic analysis of this phenomenon in flies, showing that the population of sequences derived from known miRNAs varies significantly less in the 5' ends compared to the 3' ends [18]. Furthermore, they showed that the observed 5' precision is not caused by imprecise processing by the two endonucleases Drosha and Dicer, but by an event selecting precise 5' ends at or after the 2'-O-methylation of the 3' end and Argonaute2 loading of the miRNA guide strand. These results have yet to be confirmed in a systematic way in organisms other than flies, so we investigated whether the results could be confirmed by our data.

## Results and discussion

### Data

Small RNA fractions were obtained from tissue samples of breast cancer (BC), normal adjacent breast tissue (BN), and two teratomas (CRL-7826 and CRL-7732), see Methods for details. Using 454 pyrosequencing [19] we obtained between 64894 and 302556 sequence reads from each sample. For BC, RNA up to a length of 100 nt. was extracted with the aim of identifying miRNA precursors as well as the mature product. No such precursors were found (data not shown), so for the remaining samples an upper size limit of 40 nt was used. All analyses of known miRNAs were performed with reference to miRBase 10.1 [4,5] unless otherwise stated.

### Sequence processing

#### Using a hidden Markov model for cDNA-insert recognition

**Figure 1:**
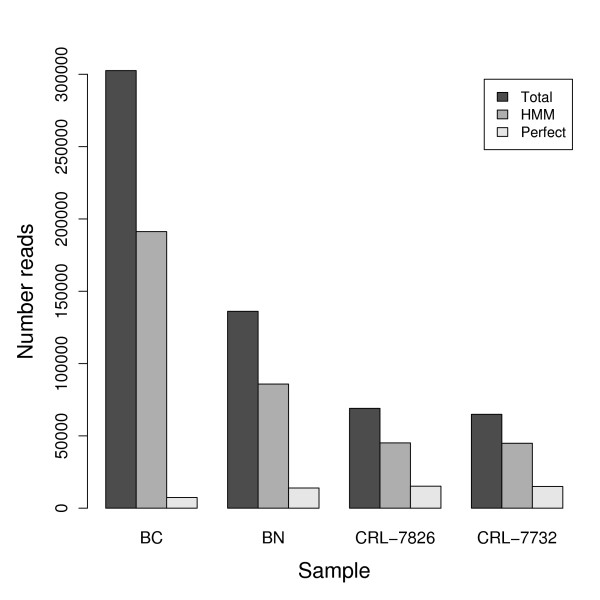
**Sequence recovery**. Extracting the actual cDNA insert from a sequencing construct. The dark grey bars show the total number of reads in each of the four samples. Medium grey bars show the number of reads where the insert could be reliably recognized using an HMM, light grey bars show the number of inserts recognized by perfect string matching to the expected flanking region. Though a sizable fraction of the raw data is lost due to errors in flanks in all cases, the HMM approach recovers a much larger part of the data.

A common step in many sequencing approaches is the ligation of short flanks of known sequence to the ends of the cDNA. These flanks must subsequently be identified and removed from the final sequence reads before analysis. Due to errors in both the production/ligation of these flanking sequences and in the sequencing reaction itself, these flanks may not always appear perfect in the final reads. The simplest approach for identifying the flanks is to only accept sequences that match perfectly to the expected flanking sequences, but this may potentially lead to a large loss in the amount of data available for further analysis. In our case, the flank regions were often imprecise, and only up to 24% of the reads would pass such perfect matching criteria in the different samples (see Figure 1). Therefore we clearly needed a way to identify the flanks correctly despite some irregularities in the sequences. Simple regular expressions or rule-based methods can be used but depend on the incorporation of prior expectations into the procedure, e.g. the position or number of expected errors. We found that even allowing two errors in a flank sequence of length 24 did not allow for robust recognition, and allowing higher error rates made the flank recognition too degenerate to be reliable. To circumvent these problems, we instead applied a probabilistic approach, by training a hidden Markov model (HMM) [20] to recognize the flanking sequences. The HMM was based on an initial model corresponding to the expected flanking sequences, with low probabilities for errors. A random subset of the data was used to train this model (unsupervised learning), letting the model automatically adjust to common variations in the flanks, so that the final model reflects the actual, observed data. By using the trained model and a suitable score cutoff we could reliably recognize the flanks for at least 63% of all sequences in each sample, see Figure 1. This approach increased the amount of usable reads by between a factor of three and 26 in the different samples. Thus an HMM offers a simple approach to drastically increase the amount of recognizable sequence inserts in the light of noise from flank ligation and sequencing.

#### Mapping the reads

**Figure 2:**
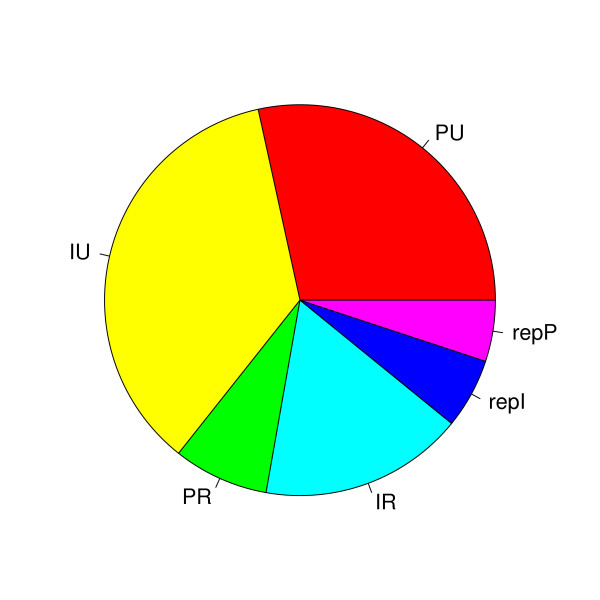
**Mapping precision**. The effect of allowing imperfect and/or multiple mappings of sequence reads. The pie chart shows the fraction of reads that map perfectly to only one unique place in the genome (PU), map imperfectly, but still to one unique place in the genome (IU), map perfectly but redundantly two to five places in the genome (PR), map imperfectly and redundantly (IR), or map repetitively, more than five places, either perfectly (repP) or imperfectly (repI). The majority of reads map uniquely to one place in the genome, both for perfect and imperfect matches.

The process of mapping the reads back to the genome is challenged both by reads mapping to multiple places in the genome, and by differences between read and the genome sequence. Such differences may occur both due to natural variation such as RNA-editing and SNPs, or more commonly errors in the sequencing process. To consider only perfect matches can therefore lead to an unacceptable loss of data. Based on these considerations, and the observed error rate in the flanks, we chose to record matches with sequence identity (mismatches and indels) as low as 90% between the read and the genomic sequence. For each read, we kept only the best match(es), i.e. those matches with the minimal number of mismatches and/or indels. To avoid any ambiguities in the mapping that heuristic algorithms such as BLAST [21] might introduce, we used the non-heuristic suffix-array based program Vmatch [22]. When using a low sequence identity cut-off, one might expect a high number of reads mapping randomly to multiple places in the genome, adding more noise than information to the data. But as can be seen in Figure 2, the majority of both perfect and imperfect matches mapped to unique places in the genome.

Given the short length and functional redundancy of miRNAs, it is not surprising that many known mature miRNA sequences map to more than one place in the genome. Of the 564 human mature miRNA sequences in miRBase 10.1, we found that 462 (82%) mapped uniquely to one place in the genome (data not shown). As a compromise between the conflicting interests of accuracy of mapping and retaining information, we chose to keep reads with up to five equally good matches. This retained 98% of the known miRNAs, and 89% of all the mapped sequence reads.

**Figure 3:**
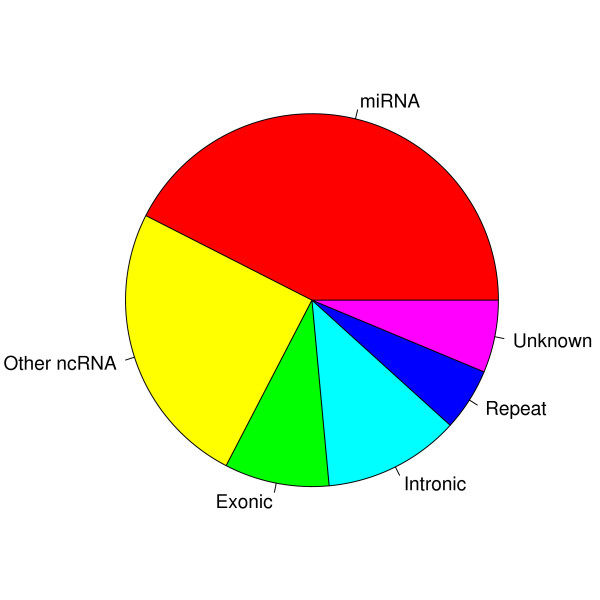
**Origin of reads**. The origin of reads after mapping to the genome, as determined by overlap with genomic annotation. As expected miRNA derived reads constituted the largest category, while (~6%) of reads were of unknown origin (no annotation on the coordinates and strand corresponding to the read). Not shown in the figure is a small number of matches to piRNAs.

To determine the origin of the mapped reads, we checked their overlap with genomic annotations, as detailed in the methods section. As expected miRNAs constituted the largest fraction of reads (Figure 3), with other ncRNAs being the second largest category. A fraction of reads not overlapping any of the known annotations was also observed. Similar to what has been reported elsewhere [12], we also observed a small number of perfect hits to piRNA sequences in all samples (data not shown).

### Differential expression in BC and BN samples

Cloning frequencies from short read libraries can be used to analyse relative expression changes between samples [17,23]. To identify differentially expressed miRNAs in the breast cancer and normal breast libraries, we used the method described by Kal *et al*. [24] with a False Discovery Rate of 0.05 [25]. This approach identified eight differentially expressed miRNAs, see Table 1. Five of these were overexpressed in breast cancer compared to normal breast tissue, including the well-known breast cancer associated miR-21 [26-29]. MiR-200b, miR-200c and miR-23a have similarly been reported to be overexpressed in cancer cells [28,30,31], consistent with our findings. Let-7a, which we found to be highly overexpressed in breast cancer, have in other studies been reported to have low expression in cancer cells [26,27,29], though the expression level has also been shown to vary between specific tumor subtypes [32], underscoring the complexity of miRNA regulation in cancer biology.

**Table 1:** Differentially expressed miRNAs in BN and BC.

miRNA	BN	BC	Fold change
mir-200b	22.8 (1)	27122.2 (2325)	1189.6
mir-200c	45.5 (2)	44072.2 (3778)	968.6
mir-21	22.8 (1)	15363.4 (1317)	673.8
mir-378	68944.3 (3027)	466.6 (40)	-147.8
let-7a	2186.5 (96)	50313.2 (4313)	23.0
mir-320	136180.4 (5979)	19376.4 (1661)	-7.0
mir-23a	11319.9 (497)	44748.8 (3836)	4.0
mir-22	25646.3 (1126)	7150.9 (613)	-3.6

MiRNAs significantly differentially expressed in normal breast (BN) and breast cancer (BC) samples, with False Discovery Rate of 0.05. Columns show the miRNA id, the observed expression in BN and BC as parts-per-million (ppm) and raw read counts in parentheses, and the relative fold change. The ppm values were based on the total number of 19 – 24 nt long reads in each library. Five miRNAs were found to be overexpressed in BC compared to BN (positive fold change), while three were underexpressed (negative fold change).

Among the miRNAs overexpressed in the normal breast compared to breast cancer samples, miR-22 has previously been reported as highly expressed in mammary progenitor cells [33]. Our findings are therefore consistent with previous reports, as well as adding new miRNAs to the repertoire of miRNAs showing different expression profiles for breast cancer versus normal breast samples.

### Identifying new miRNAs

#### Using an SVM for miRNA recognition

To identify new miRNAs in the data, we first predicted the secondary structure around a genomic match using RNAfold [34-36]. The structure prediction was done in asymmetrical windows of 15 bases to one side of the match and 60 to the other. These window lengths were chosen as the combination that generated hairpin structures for most of the known miRNAs (data not shown).

The predicted structures were then scored using an SVM trained to recognise miRNA precursor hairpins, an approach that has previously been used successfully for miRNA discovery [37-41]. Our SVM was trained on 15 different sequence and structure features, describing both the mature miRNA and its precursor (see Lindow *et al*., 2007 [42] and Methods for details). The SVM was trained using known miRNAs from miRBase [4,5] as positive examples, ensuring that miRNA-family members were kept together to avoid overfitting. In generating the negative training set, we wanted to mimic the actual task that the final SVM would be presented with: Separating true miRNAs from (fragments of) various other transcripts present in the sequencing data. We therefore sampled the negative set from a combination of sources: mRNA, non-miRNA ncRNA, and random genomic locations. To make the SVM more specific for distinguishing between genuine miRNA hairpin structures and miRNA-like structures we constrained the sampled structures by requiring that their sequence/structure features be within specific quantiles of the distributions observed for known miRNAs (detailed in Methods).

By training on these sets, we obtained a sensitivity of 80% and a specificity of 98% on an independent test set. Measures of sensitivity and, in particular, specificity, are of course completely dependent on the test data used. Given the diffculty of our training and test sets, we expect the specificity on the actual data to be higher. A high specificity is particularly important in a HT analysis setting, where even a seemingly good specificity may generate many false positives.

#### Determining expression requirements

The use of imperfect and non-unique matches increases the number of mappings to the genome, and therefore also the risk of generating false predictions. To take this into account, we examined how to incorporate the different types of matches into an expression requirement for novel miRNA loci. There is some variation in the exact mature miRNA excised from a particular miRNA precursor [12] (discussed below), so to evaluate expression we generated overall loci of the genomic matches, merging overlapping sequence matches into the same potential new miRNA. To avoid 'locus-walking', i.e. sequentially overlapping matches expanding a locus beyond what is reasonable for a mature miRNA, we restricted these loci to two-base overhangs compared to the match representing the most abundant read (see Methods for details).

**Figure 4:**
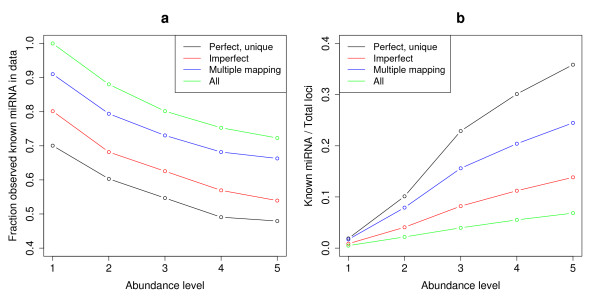
**Recovery of known miRNA, and fraction of known miRNA in the data**. (a): Recovery of known miRNAs with different matching criteria and abundance requirements. MiRNA fractions are given with respect to the total number of miRNA loci observed in the data. Abundance levels are minimum expression threshold for a locus. (b): Fraction of known miRNA to total number of loci in the data, at different match criteria and (minimum) abundance levels. Perfect, unique: Allow only reads that match the genome perfectly, and in one place only. Imperfect: Also allow reads that match imperfectly (down to 90% identity), but still in only one position. Multiple mapping: Allow only reads that match perfectly, but up to five places in the genome. All: Allow both perfect and imperfect matching, and mapping up to five places.

Figure 4a shows which fraction of the total possible observed miRNAs (the 267 known miRNA for which there is evidence in any of the samples) were observed at different expression levels, and using different matching and mapping criteria. As expected, most miRNAs were recovered by allowing all criteria and having low expression requirements. The redundant mappings were more important for miRNA recovery than imperfect matches, suggesting that most miRNAs were represented by at least one perfectly matching read.

Since the aim was to identify new miRNAs, we explored the ratio between recovered known miRNAs and the total number recovered loci at different expression thresholds (Figure 4b). The greatest increase in this ratio was observed when going from a threshold of two to three reads for a locus, with perfectly matching reads generally having higher ratios.

To balance high recovery of miRNAs with the greater miRNA/total loci ratio obtained by requiring perfect matching, we chose to require at least one perfectly matching read for a candidate miRNA locus, and a minimum total expression (perfect or imperfect matches) of three reads. The reads could be mapped either uniquely or redundantly. This gave only a 2% loss in recovered known miRNAs compared to not requiring any perfect matches, but a three-fold increase in the miRNA/total loci ratio. All loci that passed these criteria were considered likely miRNA candidates, but for a locus to be considered a reliable de facto miRNA we additionally required that perfect matching reads were observed in at least two tissues.

#### Pipeline results

Combining the SVM scoring and the expression criteria described above, yielded 20 candidate miRNA loci, with expression ranging from the minimum requirement of three up to more than 1400 reads (see Tables 2 and 3). Two of these loci represented potential new precursors for already known miRNAs. Eleven of the loci were represented by perfect mapping reads in at least two tissues, and were thus considered real miRNAs. While writing this paper a new version of miRBase was released, now including six of these miRNAs (mir-1180, mir-1271, mir-1287, mir-1296, mir-1301, and mir-1908). The remaining new miRNAs and the additional candidate loci (Additional file 1) are described below.

**Table 2:** Novel miRNAs and miRNA candidates.

Locus ID	Coordinates	Sequence	Status
12783	chr13:23634608–23634629(+)	TCTGCAAGTGTCAGAGGCGAGG	miRNA
19011	chr16:3870478–3870503(-)	GGCGGCGGCGGCGGCGGAACGG	miRNA
41039	chr5:92982172–92982192(-)	TGACAGCGCCCTGCCTGGCTC	miRNA
49828	chr9:96612080–96612101(+)	GAGAGCAGTGTGTGTTGCCTGG	miRNA
53356	chrX:151975564–151975586(-)	CGGCGGCGGCGGCGGCGGACGGG	miRNA

52195	chrX:49662029–49662050(+)	TAATCCTTGCTACCTGGGTGAG	alt
37600	chr4:17057782–17057802(-)	TCGAGGAGCTCACAGTCTAGT	alt

6219	chr10:97814116–97814137(-)	TTCAGCCAGGCTAGTGCAGTCT	cand
19702	chr17:59060613–59060634(+)	ACTGGCTTGTGGCAGCCAAGTG	cand
21361	chr17:15095708–15095729(-)	TGCTGGGGGCCACATGAGTGTG	cand
23602	chr19:764627–764645(+)	TTGGCCATGGGGCTGCGCG	cand
25697	chr2:11825070–11825091(+)	TAATGGCCAAAACTGCAGTTAT	cand
32226	chr22:49459945–49459967(+)	CCCGGGGCCAGCGCCGTGGTCGT	cand
52275	chrX:128945787–128945807(+)	CGGCGGGCGGCGGGGCGGGGC	cand

Columns show the locus ID, genome coordinates, sequence, and miRNA status: miRNA (submitted to miRBase), alt (candidate alternative precursor for known miRNA), or cand (candidate miRNA). The most highly expressed read for a locus is shown. Only loci that have not been included in miRBase yet are shown.

**Table 3:** Annotation of the miRNA candidates.

Locus ID	Annotation	Gene	RefSeq	Expression	Phastcons	Status
21019	Intron	-	AC124066.2	1,12,2,1	0.57	miR-1180
38843	Intron	ARL10	-	0,0,3,2	0.99	miR-1271
6150	Intron	C10orf33	-	4,0,1,4	1	miR-1287
6746	Intron	JMJD1C	-	1,4,2,1	1	miR-1296
27738	Intron	DNMT3A	-	3,12,7,4	0.99	miR-1301
8884	intron	FADS1	NM_013402	2,0,9,2	0.34	miR-1908
37600	repeat	-	-	136,933,146,220	0.03	alt.miR-151
52195	intron	CLCN5	NM_000084	0,4,0,0	1.00	alt.miR-500
12783	intron	PATA13	NM_153023	1,1,3,0	0.01	miRNA
19011	repeat	-	-	8,2,0,0	0.98	miRNA
41039	exon	C5orf21	NM_032042	0,2,1,0	0.46	miRNA
49828	intron	ONPEP	NM_032823	2,1,4,1	0.00	miRNA
53356	intron	PNMA5	NM_052926	7,3,0,0	0.03	miRNA
6219	intron	AK091396	AK091396	0,3,0,0	0.08	Cand
19702	intron	MAP3K3	NM_203351	84,0,1,9	0.08	Cand
21361	intron	PMP22	NM_000304	3,0,1,0	0.06	Cand
23602	exon*	PRG2	NM_002728	0,0,3,0	0.60	Cand
25697	intron	LPIN1	NM_145693	0,3,0,0	0.02	Cand
32226	exon	SHANK3	NM_001080420	3,0,0,0	0.99	Cand
52275	intron	BCORL1	NM_021946	3,0,0,0	0.99	Cand

Columns show locus ID for the putative miRNAs, annotation, gene name and RefSeq accession (where applicable), expression (read counts), and conservation. For locus 6219, where no proper gene name or RefSeq is available, GenBank accession is given instead. 'Expression' shows the raw read count for BN, BC, CRL-7826, CRL-7732, and is summed for all reads in the locus. 'Phastcons' is the average Phastcons score [52-54]. 'Status' shows if a locus is a miRNA or a candidate locus. The names of miRNAs that have already been included in miRBase are shown, with 'alt.miR-NNN' designating a predicted alternative precursor for miR-NN. *Anti-sense.

#### Novel miRNAs

None of the five remaining novel miRNAs were found to be part of a cluster (no other miRNAs within 10 Kb up- and downstream). Expression and conservation for these loci was generally low, probably reflecting that most highly expressed or conserved miRNAs have been identified by now. Three loci were intronic (12783, 49828, 53356), one of these (53356) overlapping repeat annotation as well. In addition to being intronic to one gene, locus 53356 was found to also overlap the 5' UTR of an antisense gene (*PNMA3*, [Genbank: NM_013364]), suggesting that antisense transcription might play a part in regulation of these overlapping genes.

One locus, 19011, only overlapped repeat annotation, but was part of a ~600 base pair highly conserved block, which might be transcribed as part of the 5' UTR for the nearby gene *CREBBP *[Genbank: NM_004380]. Two mRNA ([Genbank: U47741], [Genbank: U85962]) encompassing the region seem to confirm this. The repetitive CGG unit of the mature sequence was also found in the sequence of locus 53356 and candidate locus 52275.

The fifth new miRNA, locus 41039, overlapped coding exon annotation. Approximately 75 bases downstream of this locus an evolutionarily conserved secondary structure is predicted by EvoFold [43], indicative of other ncRNA or structure based regulation in the area.

#### Additional candidate miRNA loci

The remaining seven candidate loci were all represented by at least three reads, but did not fulfill our expression requirements (expression in at elast two tissues) for a reliable new miRNA. Additional data will be required to confirm these as true miRNAs. Five of the candidate loci were intronic (6219, 21361, 25697, 19702, 52275), with three of them overlapping repeat annotation as well (25697, 19702, 52275). The last two candidates (32226,23602) overlapped exons, though in the case of locus 23602 in the antisense direction. Conservation of the non-exonic candidate loci was low, with the exception of locus 52275.

#### Alternative precursors for known miRNAs

A mature miRNA sequence may be encoded by more than one hairpin precursor locus, eg. the mature miR-124 is encoded by three distinct loci. Our data suggested that two known single-locus miRNAs, miR-151 and miR-500, may be encoded by more than one locus in the genome: Reads corresponding to these miRNAs could be mapped both to their official, miRBase annotated precursor, and to alternative predicted hairpin structures elsewhere in the genome. In such cases short-read data alone cannot identify the true precursor with certainty, but the following features should be noted:

In contrast to the official mir-151 locus, the predicted alternative precursor showed only little conservation. Furthermore, while most reads map equally well both places, there were 166 reads that mapped only to the official precursor, and only three that mapped exclusively to the alternative precursor. The data therefore lends more support to the official precursor, though miRNAs derived from the alternative precursor cannot be ruled out.

**Figure 5:**
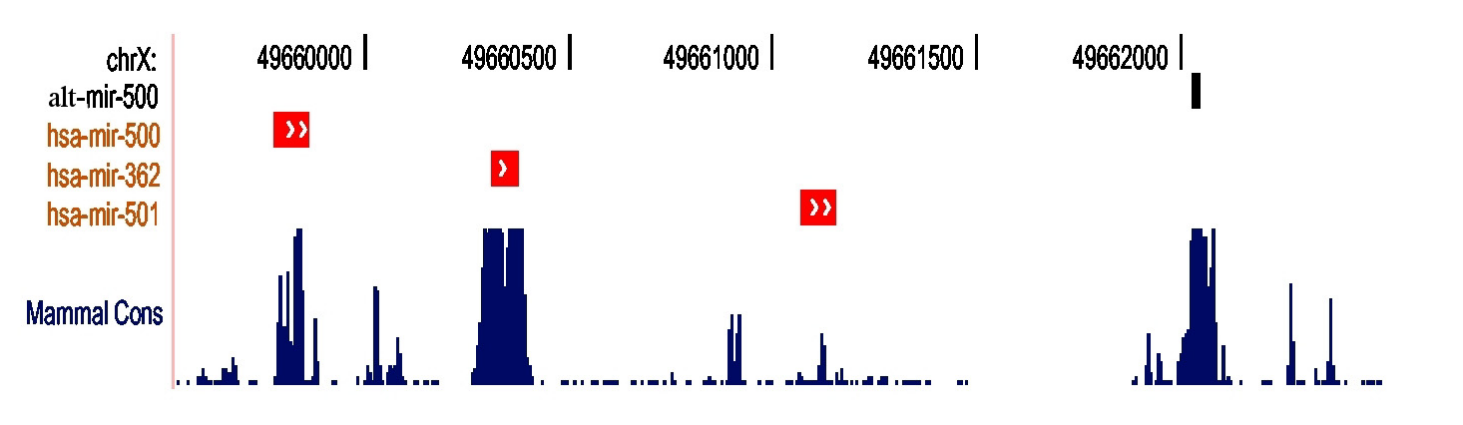
**MiRNAs and conservation near the alternative mir-500 locus**. Location and conservation of the proposed alternative mir-500 compared to the known mir-500 and nearby miRNA genes. Shown in black is the mature miR-500 sequence within the alternative precursor. The red blocks show currently known miRNA precursors, and the blue peaks show Phastcons conservation [52-54]. The mature miR-500 sequence is highly conserved in the alternative precursor. Figure generated via the UCSC genome browser [47].

The official mir-500 precursor is located within a 12 kb intronic cluster of seven annotated, conserved miRNA precursors. Our predicted alternative precursor is also located within this cluster, and similarly coincides with a peak of high conservation (see Figure 5). We therefore consider this a likely bona fide miR-500 precursor.

### Mature miRNA end precision

#### The mature miRNA 5' end is less variable than the 3' end

**Figure 6:**
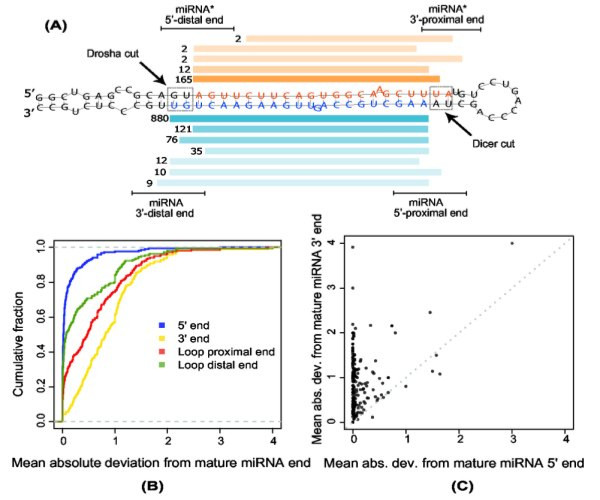
**5' and 3' miRNA end variation**. a) The figure conceptually shows how the tiling of reads in a miRNA precursor produces variation in the 5'/3' ends and loop proximal/distal ends of observed miRNA (blue reads) and miRNA* (orange reads) sequences. Expression counts are shown next to each read. In the figure miR-22 reads from the BN sample is used as example. b+c) The 219 known mature miRNAs expressed with a minimum total count of 3 in our samples. b) The cumulative distribution of mean absolute deviations from the annotated mature miRNA end. c) The mean absolute deviation of the 5' end versus the 3' end for each of the 219 expressed mature miRNAs.

To investigate the end variability of miRNAs we analyzed the 219 miRBase miRNAs for which we observed three or more reads in our data. Figure 6a shows how the miRNA precursor is processed by two different endonucleases to produce a mature miRNA product from either side of the hairpin. The first cut by Drosha is distal to the loop of the precursor hairpin, the second cut by Dicer is proximal to the loop. Comparing our reads to the annotated end positions, we calculated the absolute average deviation for 5' and 3' ends, and for loop distal and loop proximal ends compared to the miRNA precursor (Figure 6b). The 5' ends can be seen to be much less variable than the 3' ends, a difference that is highly significant (*p *< 10^-15^, Wilcoxon rank-sum). The differences between the loop proximal and loop distal ends are much less pronounced, so the observed 5' and 3' variation is not an effect of position within the hairpin.

Furthermore, the high 3' variability could not be immediately explained by 3'→5' degradation events as we found the variation to be broadly distributed on both sides of the most frequent 3' end (see Additional file 2).

Figure 6c shows the 5' versus 3' variability for individual miRNAs. Of the 219 miRNAs examined, only 7 (3%) showed most variability in the 5' end.

#### miRNA* 5' ends are also less variable than their 3' ends

**Figure 7:**
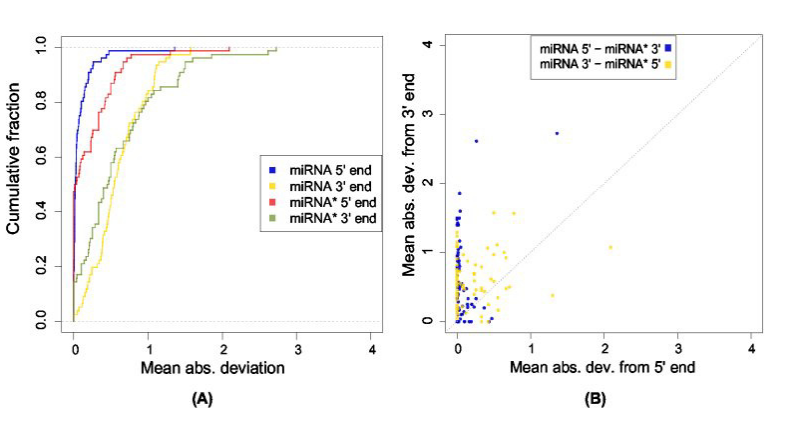
End variation of miRNA and miRNA* pairs. End variation of 79 precursors having both the mature miRNA and miRNA* region expressed. A) The cumulative distribution of mean absolute deviations from the annotated mature miRNA and miRNA* ends. B) Plotting the 5' versus 3' mean absolute deviations for all 5'/3' cleavage end pairs of the miRNA:miRNA* duplexes.

When processing the miRNA precursor, Drosha and Dicer produce a miRNA:miRNA* duplex with a fixed 2 nucleotide 3' overhang. If we assume that the population of miRNA and miRNA* sequences derived from Drosha and Dicer processing in large remains unaltered, we would expect the 5'/3' cleavage end pairs of the miRNA:miRNA* duplexes to be equally precisely defined. By analyzing reads from 79 miRNAs where the miRNA* was also expressed, we found the opposite to be true (Figure 7): the 5' end in a cleavage end pair was significantly less variable than the 3' end and this was true for both miRNA (*p *= 1.2*E *- 11, Wilcoxon rank-sum) and miRNA* 5' (*p *= 5.9*E *- 13) ends.

In summary our results on human miRNAs were consistent with those obtained for flies by Seitz *et al*. [18], and support their notion that the precise 5' ends of both miRNA and miRNA* sequences are due to a narrowing selection on a more variable sequence population produced by Drosha and Dicer.

#### miRNA loci have less variable 5' ends than non-miRNA loci

**Figure 8:**
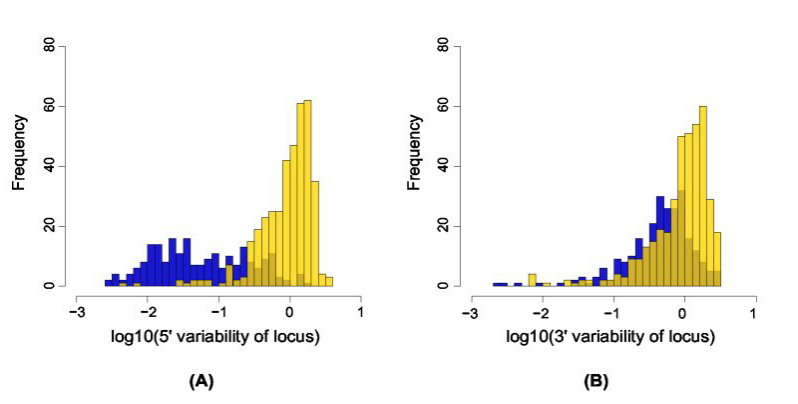
**End variation of miRNA vs. non-miRNA loci**. Distribution of A) 5' end variability and B) 3' end variability for miRNA (blue) and non-miRNA loci (yellow). Only loci with at least ten reads were used in this analysis. End variabilities of 0 are omitted from the plot.

To see whether the precisely defined 5' end is a special signature of mature miRNA sequences compared to other small RNAs in our data, we analyzed all our genomic loci with at least 10 mapped reads. The variability (average deviation from the most abundant read) was calculated as described in Methods. The distributions of 5' end variability (Figure 8), were significantly different for the two classes (Wilcoxon test, *P *< 2.2*E *- 16). The distributions of 3' end variability were similarly found to be significantly different (Wilcoxon test, *P *< 0.0033), though the distributions overlap far more (Figure 8) thus being less informative.

Together these results suggest that even though the distributions overlap, the end variation measures for a given candidate locus has some discriminatory power, and could be incorporated into a probabilistic miRNA discovery pipeline, provided there are enough reads from a given locus. Five of our putative novel miRNA loci had ten or more reads, and for these we compared the end variation to the miRNA and non-miRNA distributions. Only the locus 53356 (10 observed reads), had a 5' end deviation above what we observed for the known miRNAs. This suggests that it may not be such a reliable candidate, though having more reads available for the end deviation calculations would be preferable.

### ncRNA with miRNA-like sequence features

Kawaji *et al*. recently described a number of specific small RNA species derived from longer ncRNAs [44], in particular tRNAs, which seem to be processed in a tissue specific manner. It is interesting in this connection that when inspecting the 32 non-miRNA loci with 5' end variability less than 0.1 in our data, almost half (15) were annotated as tRNA derived, supporting the notion that these are non-random subspecies of longer tRNA transcripts. While none of these had SVM-scores indicative of a miRNA-like precursor (unsurprising given their tRNA origin), we observed that a number of high scoring hairpins were predicted in other ncRNAs, with read patterns sometimes consistent with that observed for miRNAs. For example the chromosome 17 cluster of five repetitive C/D box snoRNA U3 genes was strongly represented by a read of approximate length 22, derived from the 3' portion of the snoRNA gene (Additional file 3). Highly expressed reads from predicted hairpins were also observed in pseudo-genes for rRNAs: though a diffuse pattern of reads was observed, there were some dominant species of reads (Additional file 3). It would be interesting in future studies to see if hairpin structures inside other ncRNA genes are targeted capriciously by the miRNA processing machinery. Such ncRNA genes or pseudo genes could then easily be recruited as new miRNAs during evolution.

## Conclusion

We have analyzed small RNA sequencing data from human breast cancer tumor samples, normal adjacent breast, and two teratoma cell lines, with the aims of evaluating differential miRNA expression between breast cancer and normal adjacent breast, and to identify novel miRNAs. Several differentially expressed miRNAs were identified, adding to the growing evidence for miRNA involvement in cancer.

To identify novel miRNAs we developed a pipeline which incorporates a hidden Markov model to extract the actual cDNA from the sequencing construct, non-heuristic mapping of the reads to the genome allowing both sequence variation and mapping to several places in the genome, and a support vector machine to score predicted hairpins. Using this pipeline we identified two putative alternative loci for known miRNAs, and 11 new miRNAs. Six of these have in the meantime been independently identified by others and included in miRBase.

Inspecting the read sequences derived from mature miRNA and miRNA* pairs, we found that the 5' ends were significantly less variable than the 3' ends. Our observations support previous results in flies [18] suggesting that the low 5' variability is due to a selection on the 5' end sequences after Drosha and Dicer processing of the precursor miRNA. Furthermore, when inspecting reasonably expressed miRNA loci vs. non-miRNA loci, we found that the 5' end variability had some discriminatory power. As the depth of sequencing improves with the advent of still more powerful HT sequencing technologies, we envision that this feature might be integrated in future miRNA discovery pipelines.

## Methods

### Samples

#### Tissue

Five different human breast cancer (BC) tissue samples (about 200 mg in total) and their corresponding normal adjacent tissues (BN) were obtained from the MAMBIO repository at Herlev University Hospital, and stored at -80°C until RNA purification and fractionation. The collection of patient samples for the MAMBIO-repository was approved by the Science Ethics Committee for the former Københavs Amt and by the Danish Data Protection Agency (Datatilsynet).

#### Cell lines

The two teratoma cell lines, CRL-7826 and CRL-7732 were purchased from ATCC. The cells were grown to near confluence before total RNA extraction.

### Preparation of RNA

Tissues were ground under liquid nitrogen. Small RNA (sRNA) species smaller than 200 nt were enriched with the mirVana miRNA isolation kit (Ambion, Austin, Texas, USA). RNA from the different samples was pooled into a BC and a BN library. RNA from CRL-7826 and CRL-7732 was extracted by guanidinum isothiocyanate/phenol:chloroform extraction (Trizol). The sRNAs were then separated on a denaturing 12,5% polyacrylamide (PAA) gel. The population of miRNAs with a length of 15 – 30 and 30 – 100 bases (breast cancer samples) or length 15–40 (normal breast, teratoma) was obtained by passive elution of the RNAs from the gel. The sRNAs were then precipitated with ethanol and dissolved in water.

### cDNA synthesis

For cDNA synthesis the sRNAs were first poly(A)-tailed using poly(A) polymerase followed by ligation of a RNA adapter to the 5'-phosphate of the sRNAs. First-strand cDNA synthesis was then performed using an oligo(dT)-linker primer and M-MLV-RNAse H- reverse transcriptase. The resulting cDNAs were then PCR-amplified to about 20 ng/*μ*l using Taq polymerase.

The fusion primers used for PCR amplification were designed for amplicon sequencing according to the instructions of 454 Life Sciences. The correct size ranges (cDNA + flanks) were obtained by separate purification on 6% PAA-gels. For pool formation the purified cDNAs were mixed in a molar ratio of 3 +1. The concentration of the cDNA pool was 11 ng/*μ*l dissolved in 25 *μ*l water.

### Sequencing using 454 technology

Amplicons from all preparations were sequenced using the Genome Sequencer 20 (GS20; Roche) according to the protocol provided by Marguiles *et al*. [19], resulting in the following number of reads for each sample: BC: 302556, BN: 136139, CRL-7826: 69013, CRL-7732: 64894.

The sequence data is freely accesible and can be downloaded from . Novel miRNAs are being submitted to miRBase [4].

### Hidden Markov model

We built a profile HMM with states corresponding to the expected flank-sequences around the cDNA insert. The cDNA insert itself was modeled by a single state with fixed, uniform emission probabilities. The model was initialized with a 0.02 probability of mutation or indels in any position. A random subset of 10000 sequences was chosen and scored with the initial model. The score was calculated as , where *P*_*model *_is calculated with the forward algorithm [20], and *P*_*background *_is the probability given a uniform background model. Sequences with positive score were then used to train the final model. By inspection of the score distribution and sequences, a score cut-off above which all sequences had recognizable flanking sequences was chosen. All sequences were scored by the model, and for those that passed the score cut-off, the cDNA inserts were extracted using labels predicted by the Viterbi algorithm [20]. Inserts shorter than 18 bases were subsequently discarded, due to the diffculties of mapping such short sequences.

### Mapping sequences to the genome

We used the suffix array based program Vmatch [22] to map the read sequences to the genome requiring a minimum of 90% identity over the full length alignment. For each read we selected the set of genomic matches having maximal identity for the given read. Reads mapping more than five places with this maximal identity were discarded from further analysis.

### Annotation

Reads that had successfully been mapped to the genome a maximum of 5 places were annotated according to overlap with known annotations, in the following prioritized order:

MiRNA (Human miRBase 10.1 coordinates from miRbase [4,5,45,46]. Other ncRNA (the sno/miRNA track downloaded from the UCSC genome browser, hg18 [47-49], and the Rfam, rnaDB, joneseddy, and noncode tracks from ncRNA.org v.2.0 [50]). Exon (Known Genes exon entries from the UCSC genome browser). Intron (reads contained within the Known Genes from the UCSC genome browser, but not in exons as described above). Repeat (the repeatmasker, microsatellite, and simplerepeat tables from the UCSC genome browser).

Mapped reads not overlapping any of these features were annotated as unknown.

Reads were also mapped against human piRNAs contained in XMLpiRNAV2.zip from rnaDB.org [51] using Vmatch [22] requiring exact matches.

For assessment of conservation, the conservation scores from the 'Vertebrate Multiz Alignment & PhastCons Conservation (28 Species)' track [52-54] of the UCSC genome browser was used, and the average calculated over all base positions in the mature sequence.

### Expression analysis

The Z-test described in [24] was used to compare relative expression values for BN and BC. Only reads of length 19 – 24 were included in the analysis. Fold change was calculated based on the normalized (ppm) counts. All statistical tests were performed in R [55].

### Constructing genomic miRNA loci

To identify miRNAs among the sequenced reads, we grouped all genomic matches with read lengths between 19 – 24 nt (reads outside this range are ignored) into genomic loci based on their locations. Starting with the genomic match having highest measured read abundance, we assigned this genomic match and all matches contained within +/- 2 nt to the same locus. This procedure was repeated iteratively for the remaining genomic matches, always selecting the remaining genomic match with highest read abundance for the next locus. The genomic matches in a constructed miRNA locus represent a set of sequence variants originating from the same putative mature miRNA sequence

### Resolving miRNA precursor candidates into SVM features

For each constructed miRNA locus, we examined the secondary structure by extracting two genomic sequences around the genomic match with highest abundance in the locus. The first extracted sequence started 15 bases 5' of the match and extended 60 bases 3' of the match – the second sequence had the extension lengths reversed. Each of these was treated independently in the following analysis. Each potential precursor sequence was folded with RNAfold [34-36], and the structure processed and evaluated as described in [42], calculating a number of attributes describing both sequence and structural features. In addition to the features described in [42], we also determined the miRNA arm and the length of the longest bulge found in the calculated miRNA:miRNA* duplex.

### miRNA precursor classification

The known human miRNAs from miRBase 10.0 were used as positive examples for the SVM, excluding those where the mature sequence was annotated as shorter than 19 or longer than 24 bases. Based on the annotated mature miRNA coordinates, we constructed miRNA precursors by extension with 15 and 60 bases as described above. (Since we do not know in advance which arm of the precursor hairpin a novel miRNA will be on, this folding was done in both directions). MiRNAs that did not fold into hairpin structures using these settings were discarded. The miRBase [4] family annotation was used to ensure that family members were kept together during training.

The negative sets were made by random sampling of precursors from three different sequence sets: A) the full human genome (hg18, March 06 assembly). B) a ncRNA set made by concatenating the non-miRNA sequences from the 'rfamFull' and 'joneseddy' genome tracks from ncrna.org [50]. C) A random subset of about 9000 mRNA sequences from the 'human mRNA track', table all mrna, via the UCSC genome browser. ¿From each set 3000 – 4000 hairpin structures were sampled randomly, while requiring that the values for all SVM features were within the range observed for the miRBase miRNAs. A further 600 – 1000 hairpins were sampled from each set requiring the values to be between the 0.01 and 0.99 quantiles of the miRNA distributions, and 100 – 500 hairpins were sampled requiring values within the 0.1 and 0.9 quantiles.

We used the R e1071 library [56] implementation of an SVM with radial kernel, using ten-fold cross-validation and evaluation on an independent test set. A locus was assigned the highest score obtained by any of its reads.

### miRNA end precision

For miRBase mature miRNAs, reads mapping to the annotated mature region relaxed by +/- 4 nucleotides in both ends were analyzed. We only examined miRNAs having mapped reads with a summed expression count of at least 3. As a dispersion measure of the mature miRNA end precision we used the weighted mean absolute deviation (WMAD) with weights defined by the expression counts of the reads. Let *x*_*a *_denote the annotated mature miRNA end position (e.g. 5' end), and for each read *r*_*i *_∈ *r*_1_, *r*_2_,...,*r*_*n *_mapping to the region we denote the expression count *c*_*i *_and the end position *x*_*i*_:



The same measure was used with signed distances (*x*_*i *_- *x*_*a*_) instead to infer the directionality of the dispersion relative to the annotation. For comparisons of miRNA-miRNA* end precision, the WMAD was calculated relative to the respective sequences with highest read abundance.

## Competing interests

NT, SM, and TL are employees of Exiqon A/S, DK-2950 Vedbæk, Denmark. The remaining authors declare no competing interests.

## Authors' contributions

SN and AJ analyzed the data and wrote the paper. ML developed software to manage 454-data and analyse miRNA-structure. AK built HMM models, supervised the analysis, and helped write the paper. HF performed the surgery on the breast cancer patients. EB prepared, diagnosed and dissected the patient samples. JE cultivated teratoma cell lines and prepared RNA samples. NT, SM and TL conceived of the experimental part of the study, its design and coordination, and helped write the paper. SN is corresponding author for the computational analyses, TL is corresponding author for the experimental part of the study. All authors read and approved the final manuscript.

## Pre-publication history

The pre-publication history for this paper can be accessed here:



## Supplementary Material

Additional file 1 **Figures of expression of novel miRNA loci**. Expression is shown for all the putative novel miRNAs described in this paper. Each figure shows the genomic coordinates (top row), location of the approximate predicted precursor hairpin (second row: grey box = mature, white box = miRNA*), and all reads mapped to the region. Each bar represents one specific read. The bars are colour coded according to samples and expression, as labeled in each figure. Thick bars represent perfect matches, thin bars imperfect matches. Note that the approximate miRNA* (white box) is a computational construct, not the actual biological miRNA* expected from the locus. This file is best viewed on-screen. Click here for file

Additional file 2 **Figure of signed end variation of miRNAs**. The 219 expressed known mature miRNAs with a minimum expression count of 3. A) The distribution of mean (signed) deviations from the most frequent mature miRNA 5' end (56 miRNAs with a 5' deviation of 0 are omitted for plotting purposes, minus denotes shorter sequences). B) The distribution of mean (signed) deviations from the most frequent mature miRNA 3' end, minus denoting shorter sequences. Ten miRNAs with a 3' deviation of 0 are omitted. Click here for file

Additional file 3 **Figures of miRNA-like expression from other ncRNA genes**. Two examples of read expression patterns in predicted hairpins in non-miRNA ncRNAs. First example is within a C/D box snoRNA U3 gene (five such genes are repeated on chromosome 17). A dominant read of approximate size 22 is observed. Second example is within a 18S rRNA related pseudogene. Despite a diffuse expression pattern, there is a dominant species of read. Legend: Each figure shows the genomic coordinates (top row), location of the approximate predicted precursor hairpin (second row: grey box = mature, white box = miRNA*), and all reads mapped to the region. Each bar represents one specific read. The bars are colour coded according to samples and expression, as labeled in each figure. Thick bars represent perfect matches, thin bars imperfect matches. Note that the approximate miRNA* (white box) is a computational construct, not the actual biological miRNA* expected from the locus. This file is best viewed on-screen. Click here for file
