# Supplementary figures and images for: Identification and analysis of miRNAs in human breast cancer and teratoma samples using deep sequencing
Source: BMC Med Genomics. 2009 Jun 9;2:35. doi: 10.1186/1755-8794-2-35 (PMC2702338; doi:10.1186/1755-8794-2-35)

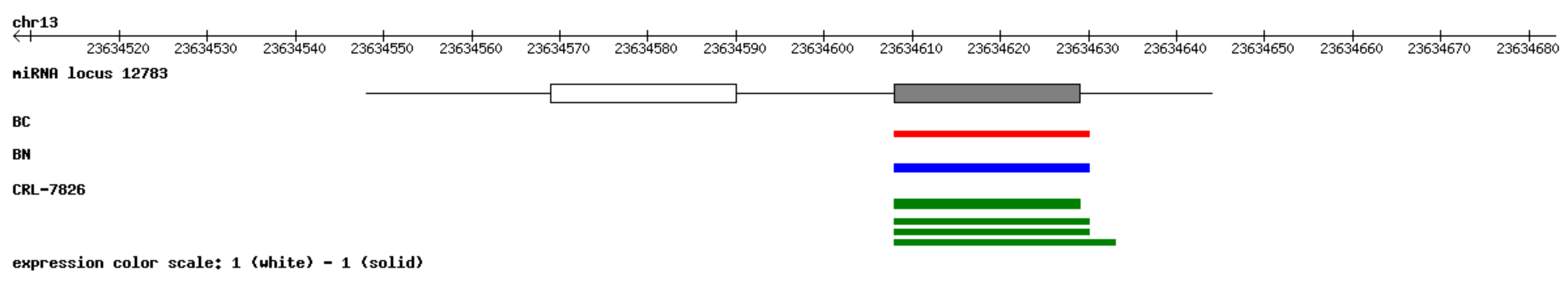

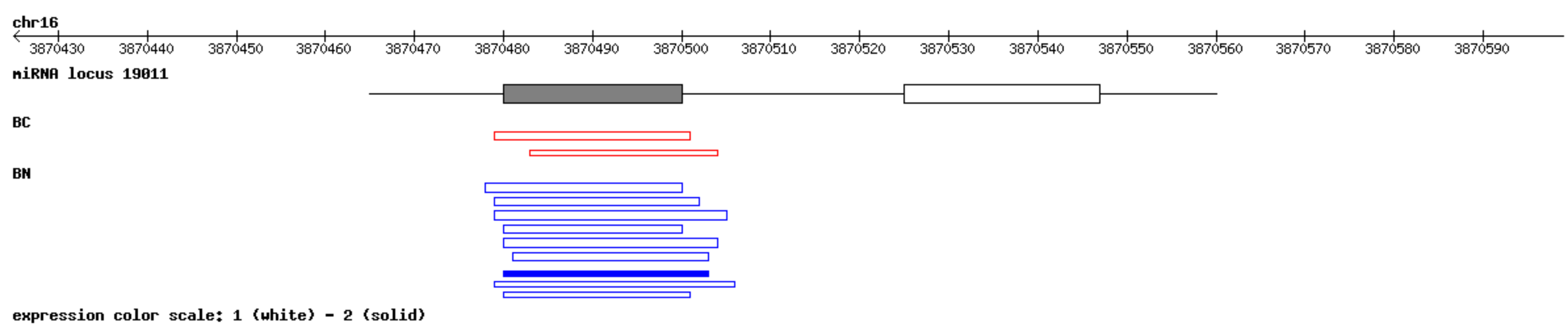

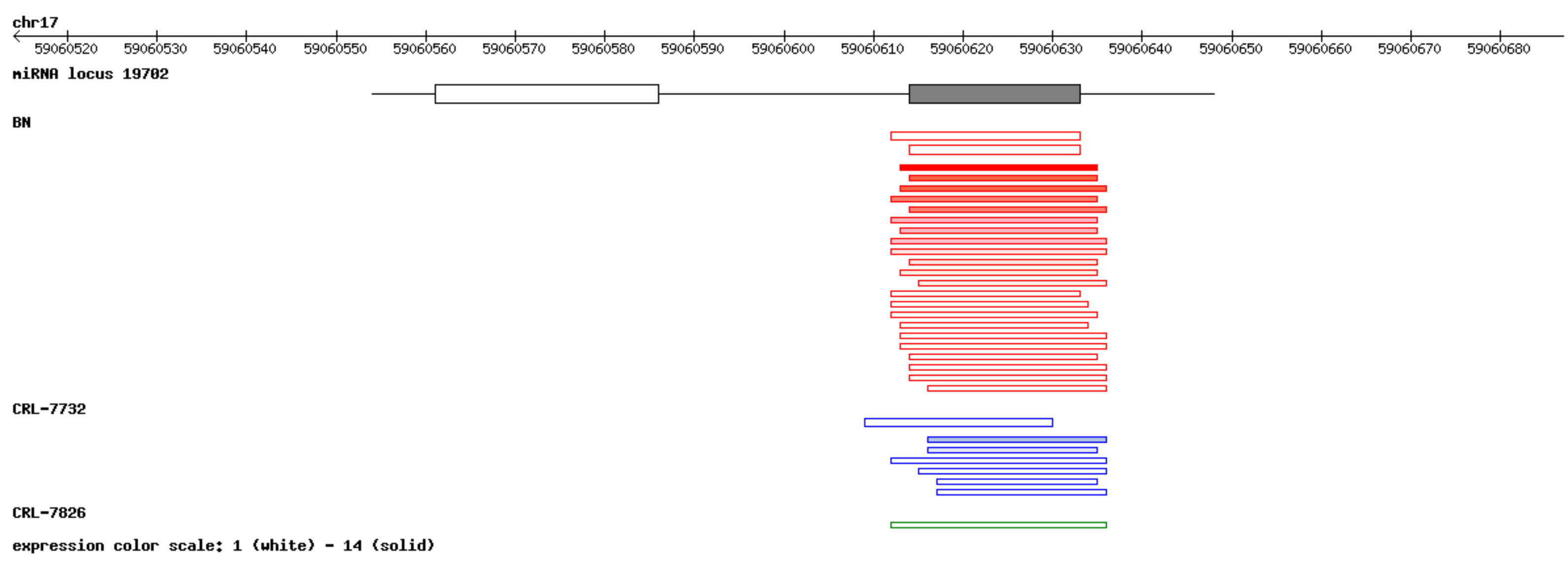

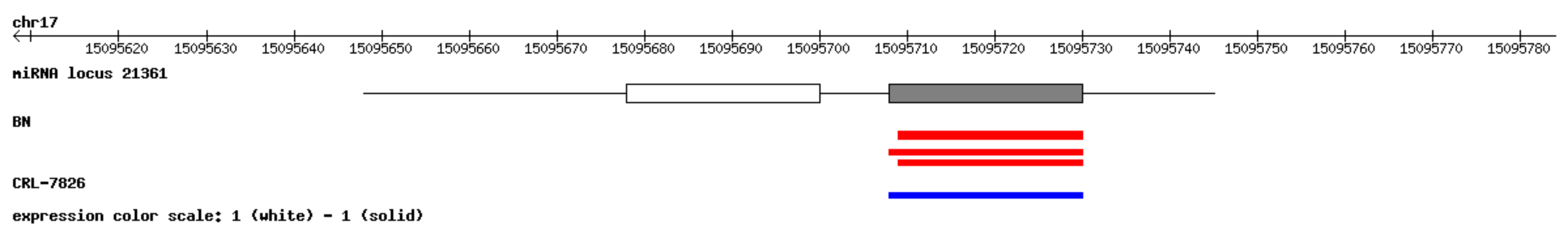

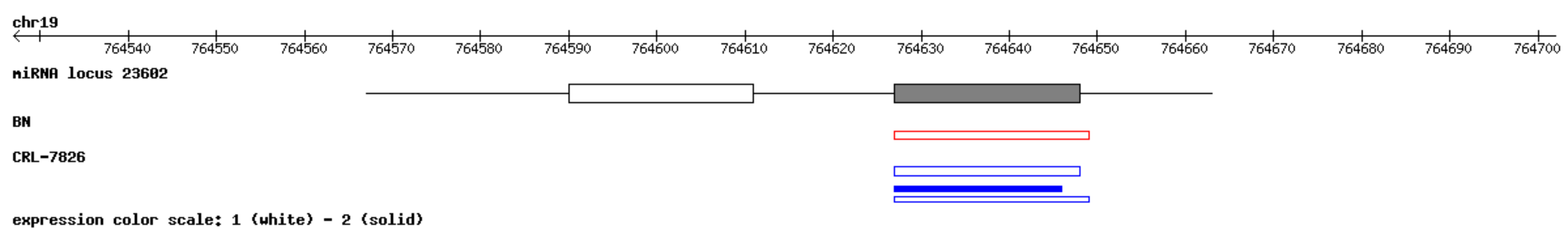

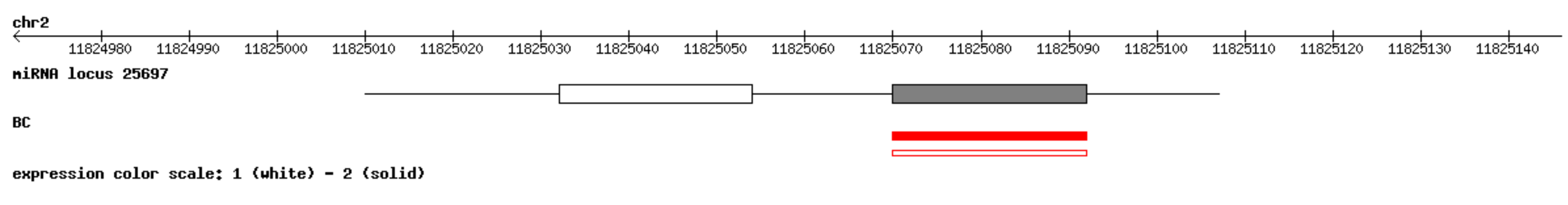

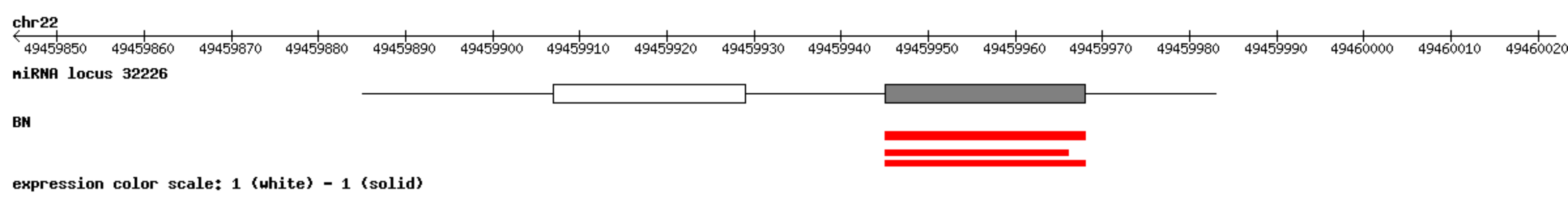

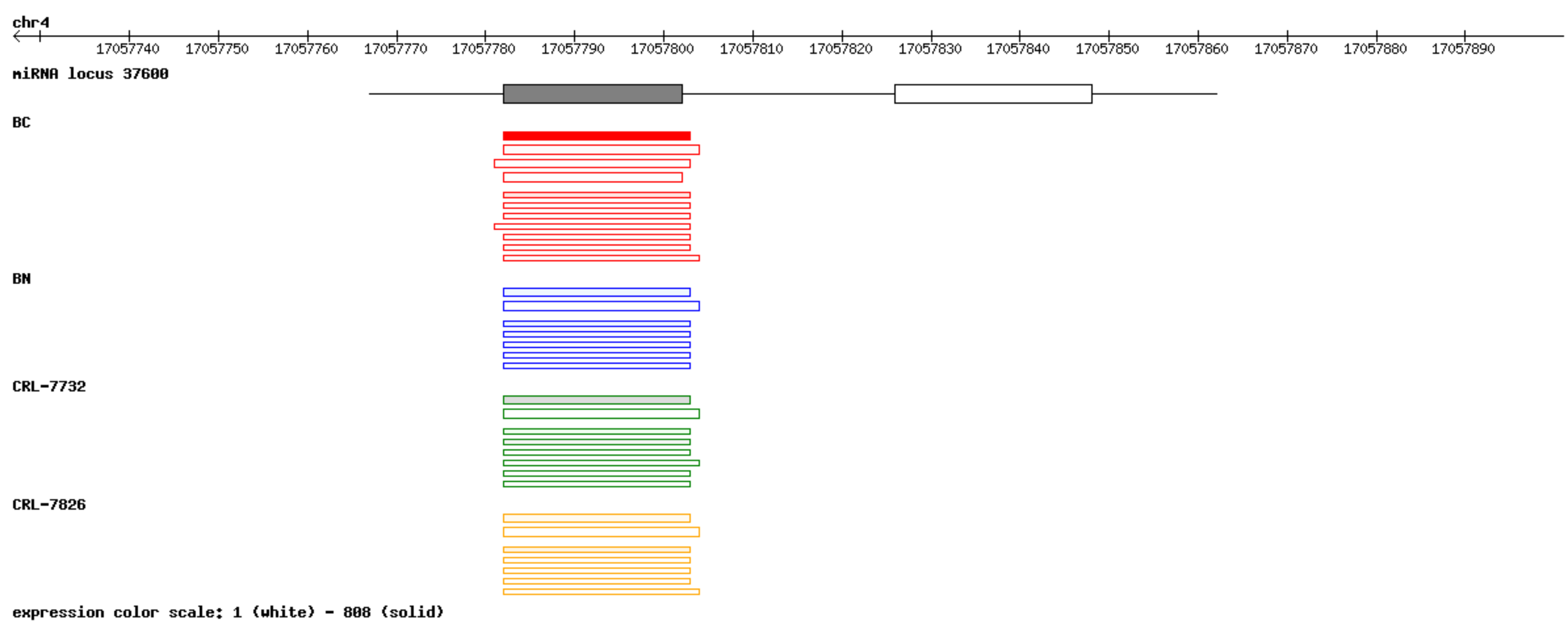

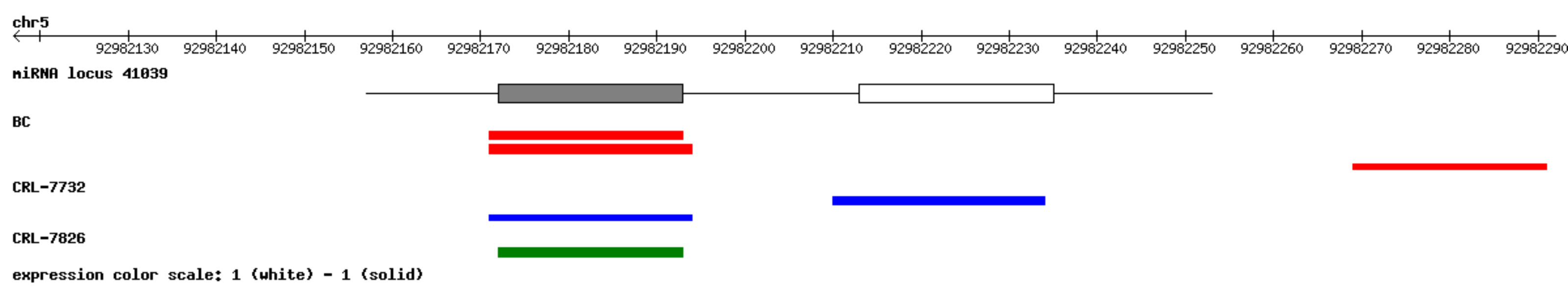

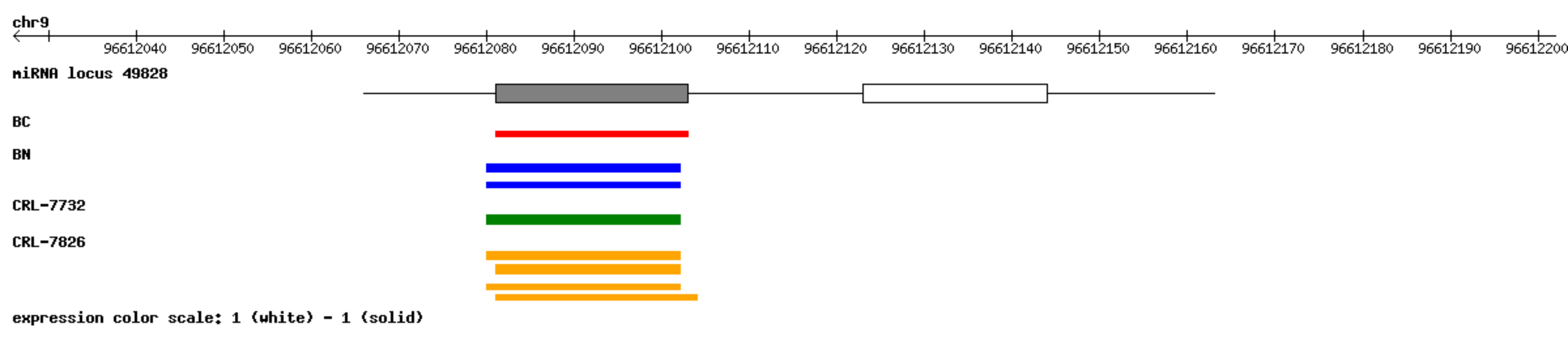

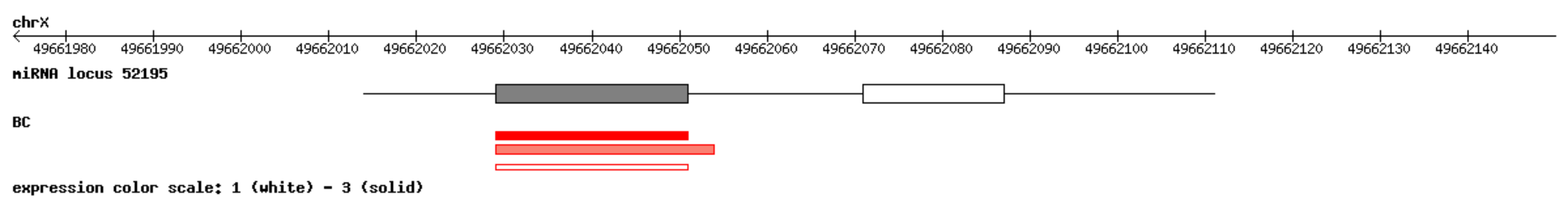

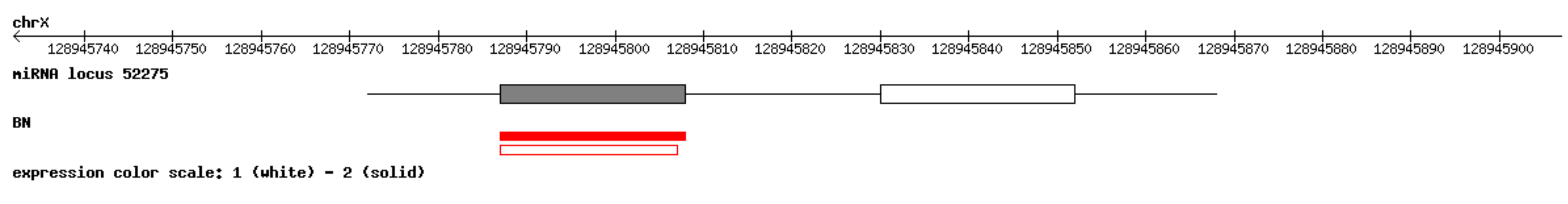

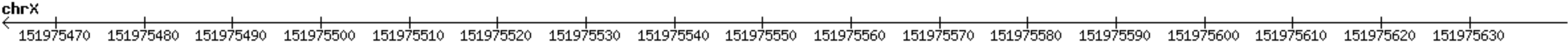

miRNA locus 53356

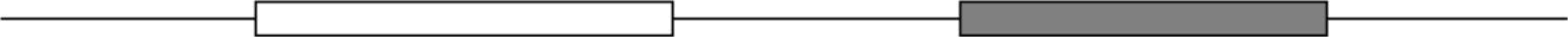

BC

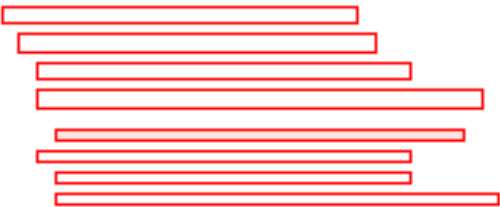

BN

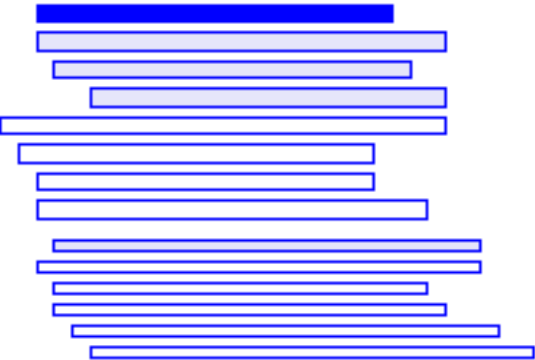

CRL-7826

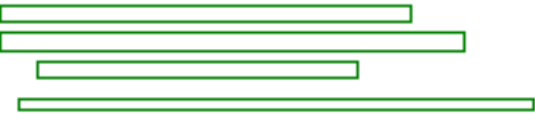

expression color scale: 1 (white) - 7 (solid)

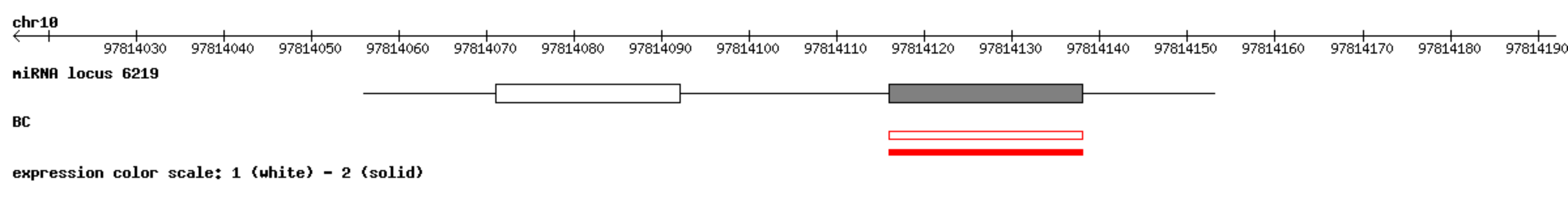

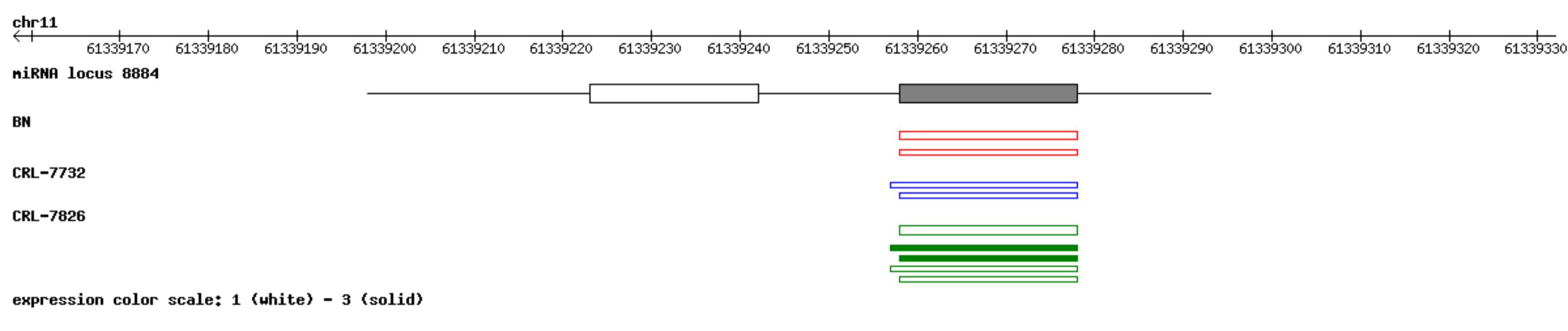

Supplement: Additional file 1 — Figures of expression of novel miRNA loci. Expression is shown for all the putative novel miRNAs described in this paper. Each figure shows the genomic coordinates (top row), location of the approximate predicted precursor hairpin (second row: grey box = mature, white box = miRNA*), and all reads mapped to the region. Each bar represents one specific read. The bars are colour coded according to samples and expression, as labeled in each figure. Thick bars represent perfect matches, thin bars imperfect matches. Note that the approximate miRNA* (white box) is a computational construct, not the actual biological miRNA* expected from the locus. This file is best viewed on-screen. [file 1755-8794-2-35-S1.pdf]

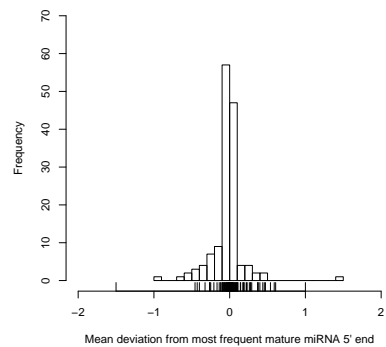

(a)

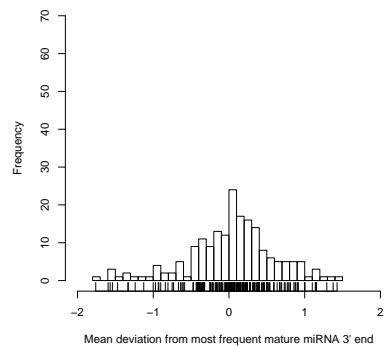

(b)

Supplement: Additional file 2 — Figure of signed end variation of miRNAs. The 219 expressed known mature miRNAs with a minimum expression count of 3. A) The distribution of mean (signed) deviations from the most frequent mature miRNA 5' end (56 miRNAs with a 5' deviation of 0 are omitted for plotting purposes, minus denotes shorter sequences). B) The distribution of mean (signed) deviations from the most frequent mature miRNA 3' end, minus denoting shorter sequences. Ten miRNAs with a 3' deviation of 0 are omitted. [file 1755-8794-2-35-S2.pdf]

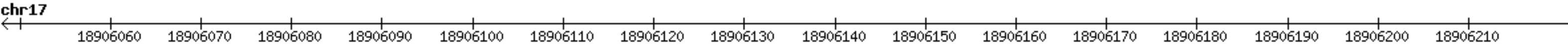

**miRNA locus 19691**

**BC**

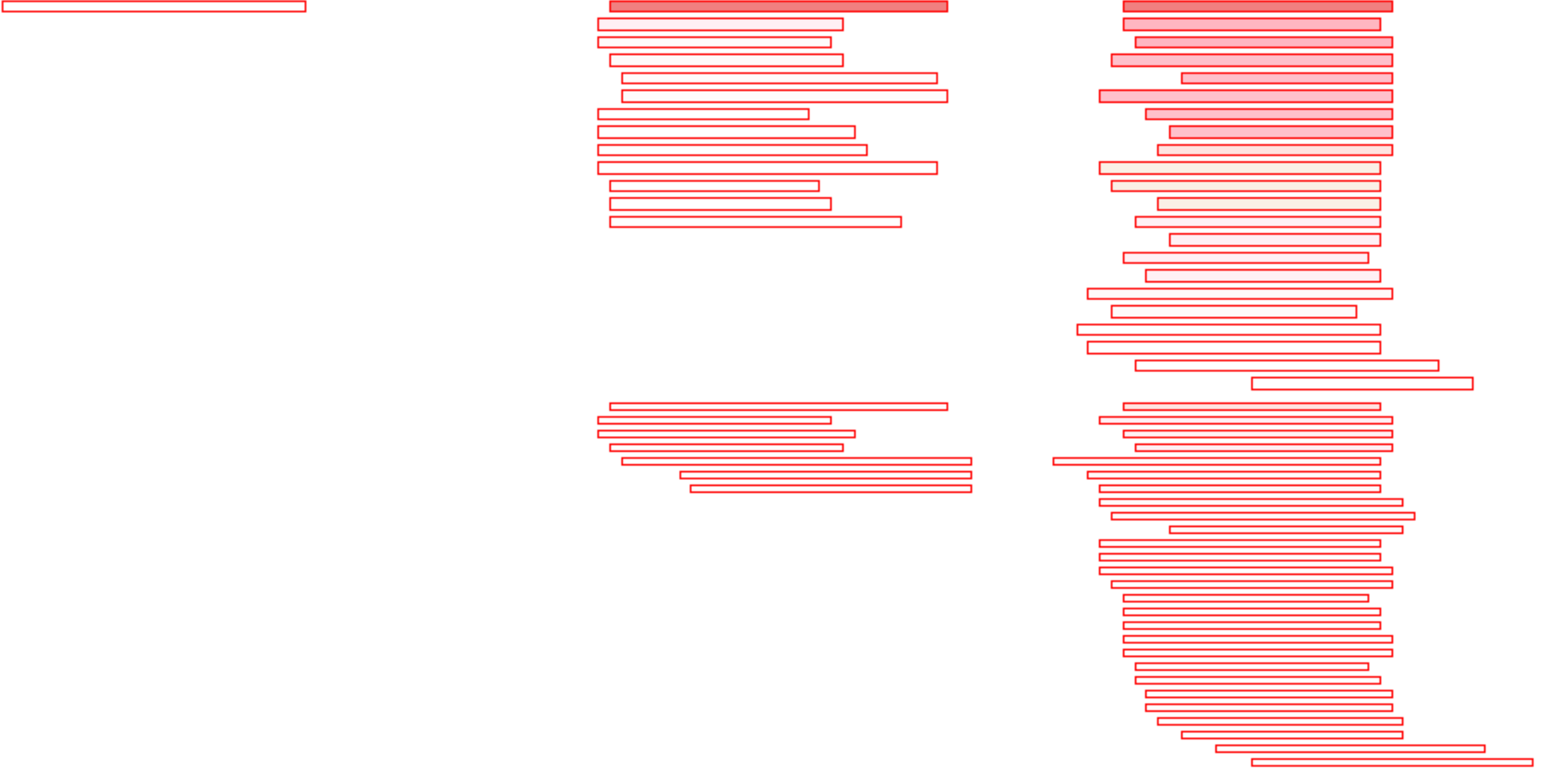

**BN**

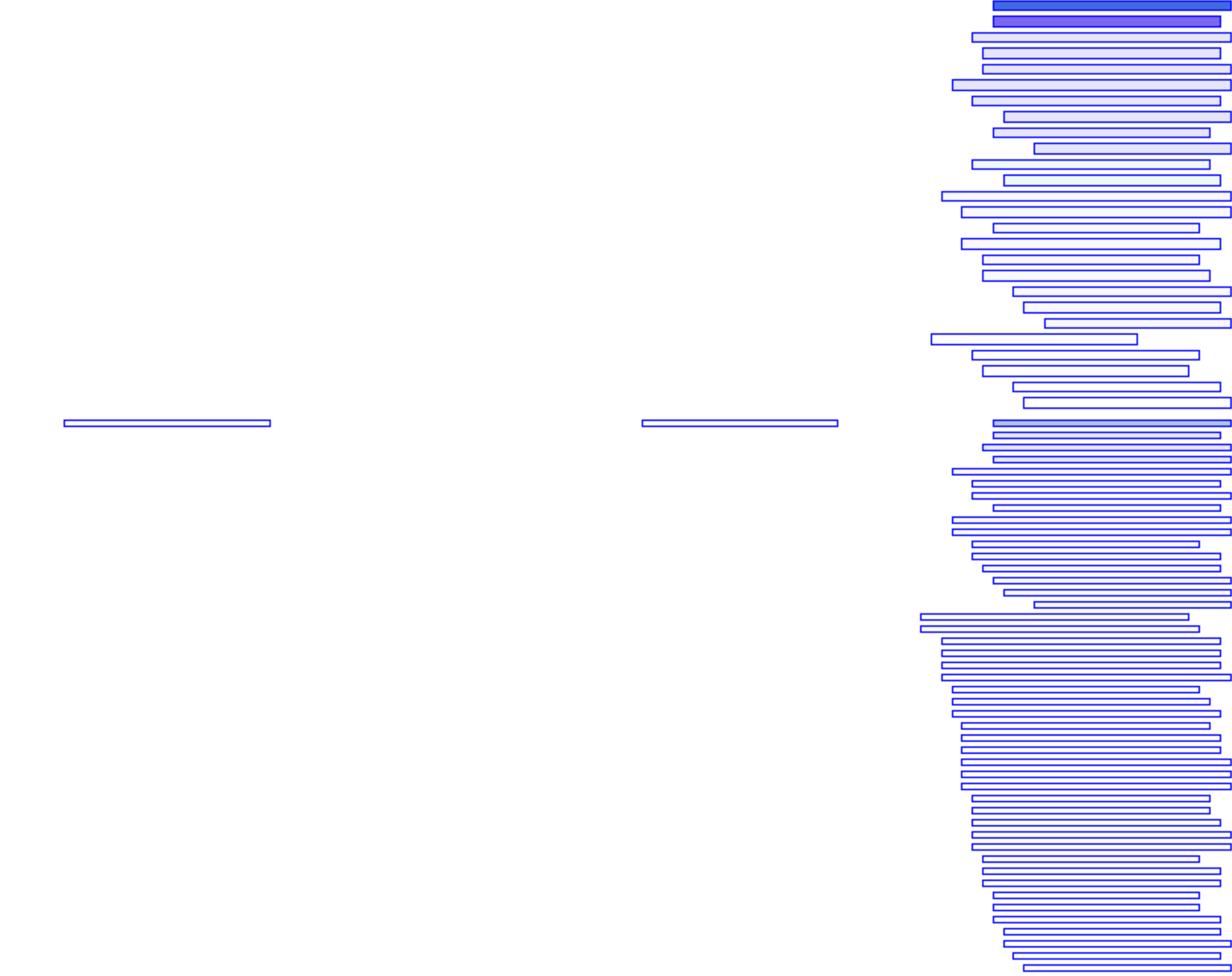

**CRL-7732**

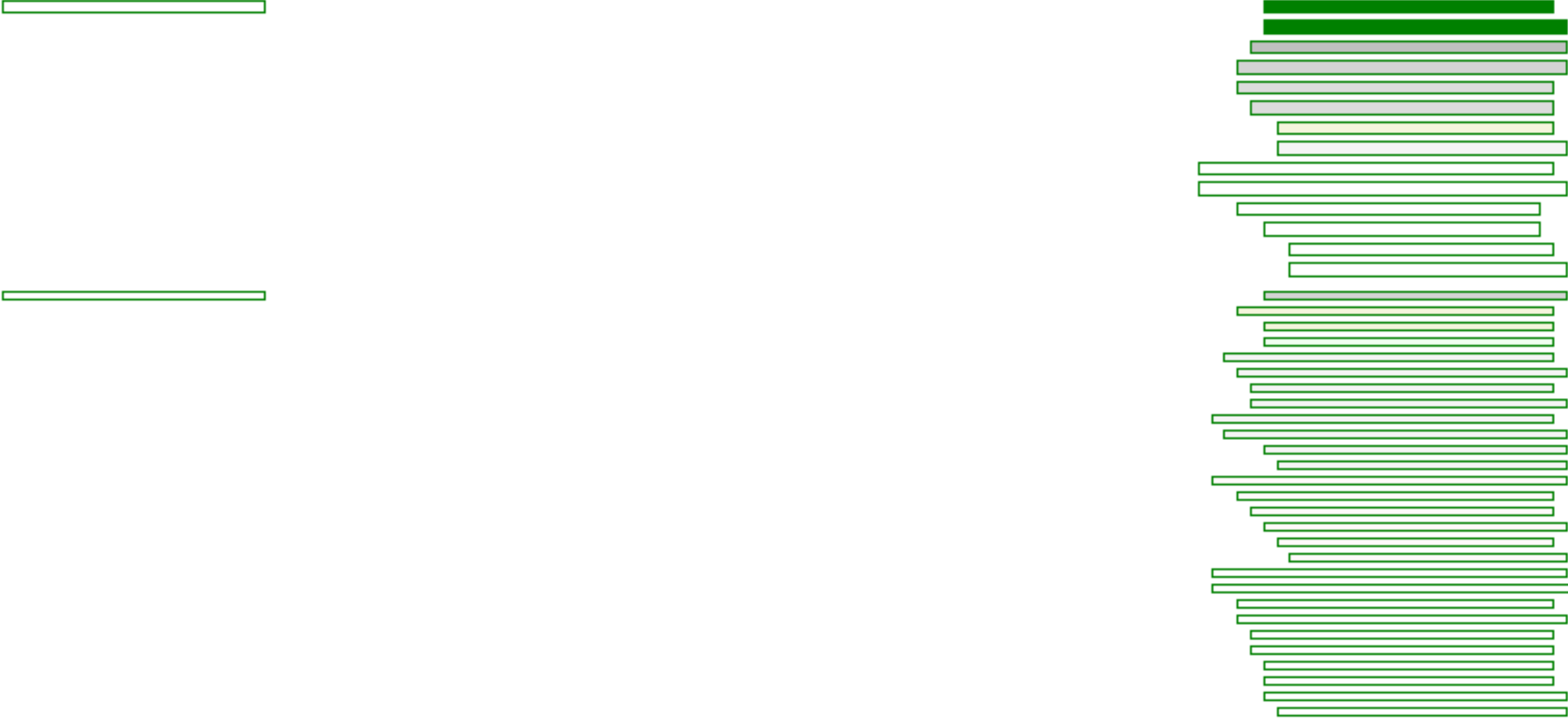

**CRL-7826**

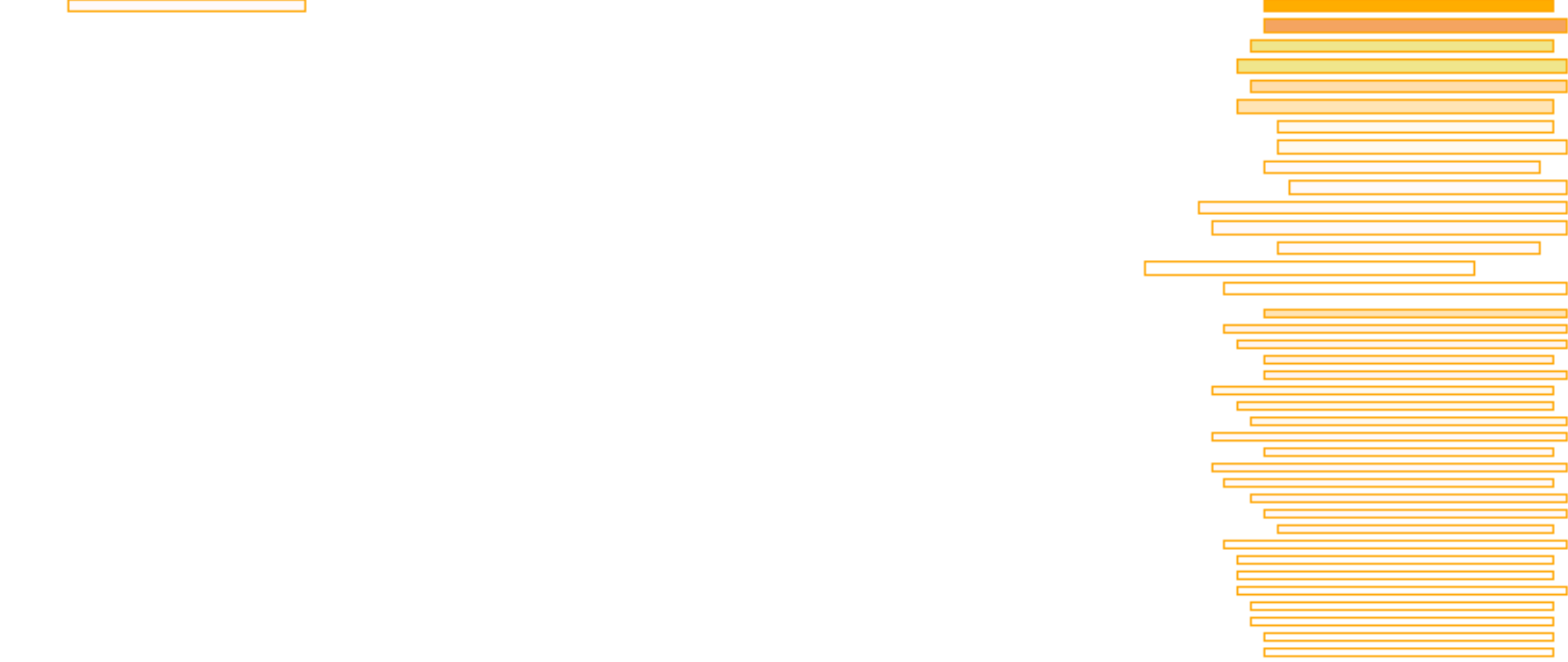

**expression color scale: 1 (white) - 53 (solid)**

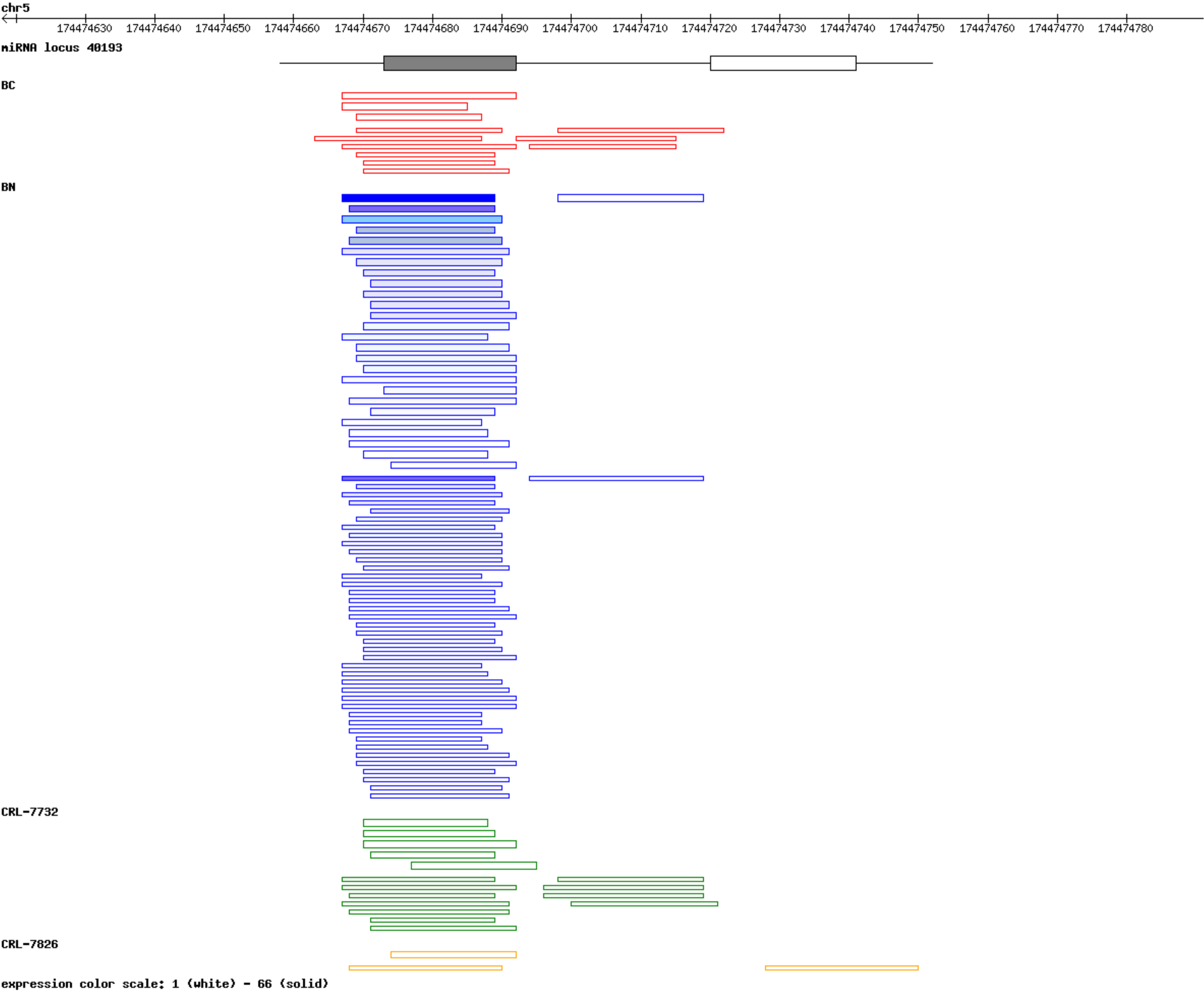

Supplement: Additional file 3 — Figures of miRNA-like expression from other ncRNA genes. Two examples of read expression patterns in predicted hairpins in non-miRNA ncRNAs. First example is within a C/D box snoRNA U3 gene (five such genes are repeated on chromosome 17). A dominant read of approximate size 22 is observed. Second example is within a 18S rRNA related pseudogene. Despite a diffuse expression pattern, there is a dominant species of read. Legend: Each figure shows the genomic coordinates (top row), location of the approximate predicted precursor hairpin (second row: grey box = mature, white box = miRNA*), and all reads mapped to the region. Each bar represents one specific read. The bars are colour coded according to samples and expression, as labeled in each figure. Thick bars represent perfect matches, thin bars imperfect matches. Note that the approximate miRNA* (white box) is a computational construct, not the actual biological miRNA* expected from the locus. This file is best viewed on-screen. [file 1755-8794-2-35-S3.pdf]
